# Perceptions and compliance with COVID-19 preventive measures in Southern and Central regions of Mozambique: A quantitative in-person household survey in the districts of Manhiça and Quelimane

**DOI:** 10.1371/journal.pone.0278439

**Published:** 2024-05-14

**Authors:** Ariel Nhacolo, Amílcar Magaço, Felizarda Amosse, Aura Hunguana, Teodimiro Matsena, Arsénio Nhacolo, Elisio Xerinda, Quique Bassat, Charfudin Sacoor, Inacio Mandomando, Khátia Munguambe

**Affiliations:** 1 Centro de Investigação em Saúde de Manhiça (CISM), Maputo, Mozambique; 2 ISGlobal, Hospital Clínic - Universitat de Barcelona, Barcelona, Spain; 3 ICREA, Pg. Lluís Companys, Barcelona, Spain; 4 Pediatrics Department, Hospital Sant Joan de Déu, Universitat de Barcelona, Barcelona, Spain; 5 Instituto de Salud Carlos III, CIBER de Epidemiología y Salud Pública, Madrid, Spain; 6 National Institute of Health, Ministry of Health, Maputo, Mozambique; 7 Faculty of Medicine, Eduardo Mondlane University, Maputo, Mozambique; University Eduardo Mondlane, MOZAMBIQUE

## Abstract

The COVID-19 pandemic has prompted countries to swiftly implement rigorous preventive measures on a population-wide scale worldwide. However, in low-income countries like Mozambique this was difficult, coupled with a generalised lack of knowledge on how the population understood and complied with these measures. This study assessed community perceptions and implementation of anti-COVID-19 measures recommended by Mozambican authorities in Manhiça and Quelimane districts, including confinement, social distancing, frequent handwashing, mask wearing, and quarantine as the key practices to evaluate. We conducted a cross-sectional quantitative survey in October 2020 and February 2021, interviewing heads of households, face-to-face. The data collected included self-evaluation of compliance and existence of handwashing facilities and face-masks in the households, aided by observations. We present descriptive statistics on perceptions and compliance at individual and household levels. Out of the 770 participants, nearly all (98.7%) were aware of Coronavirus disease, including the term COVID-19 (89.2%). Knowledge varied between districts, with Manhiça participants showing higher levels of sufficient ability to define the disease. The symptoms most mentioned were dry cough (17.8%), fever (15.7%), flu-like symptoms (14.2%), breathing difficulties (13.6%), and headache (13.1%). Participants recognized various transmission modes, including touching infected objects and inhaling infected air. Preventive measures like handwashing with soap or sanitizing hands with alcohol, wearing masks, and social distancing were acknowledged, but the understanding varied. Compliance with these measures was generally low, with fewer than half of respondents reporting adherence to them. Only 30.4% of households had handwashing facilities (of which only 41.0% had water), and masks were often limited to one per person aged 6 years or more. Community members in Manhica and Quelimane were aware of COVID-19 but had limited understanding of what the preventive measures meant, and had lower levels of compliance. Understanding and addressing the factors affecting the proper implementation of these measures is crucial for improving community adherence in preventing infectious diseases with epidemic potential.

## 1. Introduction

Since COVID-19 was declared a pandemic disease [1], different approaches to contain the spread of the virus have been adopted around the world, with countries taking stringent measures which included, among others, closing the borders, banning incoming flights, shutting down educational institutions and non-essential services, curfews, and complete population lock-down [2,3]. While the implementation of these measures in high-income countries was already challenging, in low-income countries like Mozambique they represented an incredible conundrum. Mozambique initially declared level 3 (in a scale of 1 to 4) State of Emergency, which included measures such as closing down educational institutions, interrupting visa services, limiting mobility, limiting gatherings to a maximum of 50 people and recommending a minimum distance of 1.5 meters between individuals. Later on, gatherings were limited to a maximum of 10 people and the use of masks was recommended in crowded places and public transport vehicles [4]. The Mozambican Government focused on prevention, recognising that the health care system was far from being capable of responding to a massive number of COVID-19 patients, should the pandemic reach its peak early and abruptly [4]. This was in parallel with preparation of the health care system to respond to the pandemic, which included acquisition of medicine, training of medical staff, establishment of camps for quarantine, and boosting health information system–these and other measures were detailed in the Mozambican National Plan for the Response to COVID-19 Pandemic, which included details of how the different public and private sectors would interact at national, provincial, district, and community levels [5].

However, when the restriction measures were being implemented there were reports, in the media, of crowded public transports, markets and streets, and of people not having interrupted activities such as working, or trading or travelling for the sake of their families’ livelihood, even during the harshest restriction periods [6]. For the same reasons of self and family survival, there were indications that the “stay at home” principle was a serious challenge to an important segment of the population, who lived on the basis of a daily income [7]. Little is known about whether and how the communities understood and implemented the measures recommended for preventing or reducing the spread of COVID-19 in Mozambique.

Understanding the gaps in knowledge and compliance with measures against the spread of COVID-19 in Southern and Central Mozambique would confer the opportunity to develop more effective and socio-culturally appropriate sensitization initiatives to address the need of COVID-19 prevention. This study aimed to assess the perceptions and implementation of the measures recommended by the government of Mozambique to prevent COVID-19 in rural and urban settings of Southern and Central Mozambique (Manhiça and Quelimane districts), including confinement, social distancing, hand washing, mask wearing, and quarantine as the key practices to assess. Data such as these, from both rural and urban areas and from two socio-economically different regions of the country, were crucial for informing decision-makers about ways to improve community knowledge and practices regarding prevention of COVID-19 or any other future infectious disease with epidemic potential.

## 2. Methods

### 2.1. Study design

This study was a cross-sectional quantitative household survey designed to collect data both at individual and household level, through interviews administered in-person to the heads of households or their representatives, gathering their perceptions and practices regarding anti-COVID-19 measures and in the context of their households.

### 2.2. Study setting and population

The study took place in two locations: (i) Manhiça district, a rural setting located in Maputo Province, in the Southern region of Mozambique; and (ii) Quelimane district, located in Zambézia province, in the Central region, which comprises urban and rural settings. Mozambique is divided into 11 provinces, which are generally grouped in three regions based on cultural and socio-economic similarities. The Northern region comprises 3 provinces (Cabo Delgado, Niassa, and Nampula); the Central region comprises the provinces of Zambézia, Tete, Manica, and Sofala; and the Southern region, Maputo City, Maputo province, Gaza, and Inhambane. The Southern region is the most prosperous, followed by the Central region [8]. Manhiça district is 80 km North of Maputo City, the capital of Mozambique, and was purposively selected because of the presence of the Manhiça Health Research Center (Centro de Investigação em Saúde de Manhiça—CISM). CISM has been conducting biomedical research in the district over the past 26 years, which has facilitated the implementation of the study in a context of emergency with relatively less challenges than it would have been elsewhere in rural Mozambique.

Similarly, the district of Quelimane was chosen because CISM has been conducting biomedical research there since 2018 [9]. Currently, CISM is establishing an HDSS in the district. Quelimane is located along the river Rio dos Bons Sinais, in the Southern part of Zambézia province. The district has urban and rural settings. The urban area comprises the City of Quelimane (the capital of Zambézia province), where 72% of the total 350,000 district population lives [10].

The study population comprised heads of households or their representatives that were residents in the study area, as defined by the CISM’s HDSS, i.e. those who live in a household in Manhiça or in Quelimane districts for three or more months or are entering the district with the intention to do so [11]. The survey adopted, also, the HDSS definition of household, as a group of one or more individuals who live together in the same house or group of houses, eat together, share domestic expenses, and acknowledge one of them as their head or leader [11]. The head of a household is the member who takes the most important day-to-day decisions in the household and is the reference member [11].

### 2.3. Sampling and sample size

In Manhiça, the survey used the HDSS database as a sampling frame to randomly select a sample of households that was representative at the level of Administrative Posts. In Quelimane the sampling frame was a list of households provided by the local authorities. Although the study had multiple outcomes of interest at household level, the presence of a handwashing facility with water and soap was considered as the main variable for estimating the minimum sample size of households for this study. A handwashing facility was defined as a device to contain, transport or regulate the flow of water to facilitate handwashing [12]—a commonly used proxy indicator of actual handwashing practice, which have been reported to be more accurate than other proxies such as self-reports of hand washing practices. Because there were no data on the proportion of households with a handwashing facility during the pandemic in Mozambique, the sample size was calculated to estimate a proportion of 50% with a margin of error of 5% and confidence level of 95% [13]. Thus, it was estimated that a sample size of 385 households for each district would be sufficient to estimate this proportion (a sample size calculated this way is also suitable to estimate proportions ranging from 10% to 90%) [13]. At the individual level, the main outcome was defined as the proportion of individuals who wash their hands at critical points in time, but because there were no data for the pandemic period, the sample size was calculated to estimate a proportion of 50% of people washing their hands, with a margin of error of 5% and confidence level of 95%, which resulted in a sample size of 385 individuals for each district. Thus, because the sample size for households coincided with that of individuals, the data for the two units of analysis (individual and household) were collected by asking the questions to the same respondent, i.e. one respondent per household.

### 2.4. Data collection and quality assurance

The data were collected in October 2020 in Manhiça and in February 2021 in Quelimane, using paper-based questionnaires that were verified by demographers for consistencies and completeness, and were double-entered to reduce typing errors at CISM’s Data Center. Questionnaires with errors were returned to the field for corrections. The interviewers were selected from the HDSS fieldworkers (supervisors and other well-experienced fieldworkers) who were carefully trained for this survey. The training included refreshing the training that they had previously received on biosafety measures in the context of the coronavirus pandemic, as part of CISM’s requirements. The survey questionnaire was designed to collect the following data:

**Perceptions and level of compliance with COVID-19 preventive measures**–using questions framed to capture knowledge and understanding of the disease, details of symptoms, means of transmission and prevention, definitions of preventive measures, and level of compliance with anti-COVID-19 measures at individual and household levels. Thus, questions such as “have you ever heard of coronavirus?”; “have you ever heard of COVID-19”; “if YES, what is it?”; “what are the symptoms or signs of Coronavirus or COVID-19? “; “how is this disease transmitted?”; and “how can we prevent it?” were asked. To assess the understanding of anti-COVID-19 measures, respondents were asked the following questions: “could you please explain what does social distancing mean?”; “could you explain what it means to always wear face mask?”; “what it means to avoid touching the mouth/nose/eyes?”; “what does quarantine mean?”; “what it means to avoid crowded places?”; “what it means to avoid travelling?”. This included questions for self-evaluation of compliance with the key measures considered in this study. The respondents were asked to grade themselves in a scale of 0% to 100%, where 0% meant that they did not comply at all with a specific measure, and 100% when they thought they complied completely with a given measure. Most of the questions had pre-defined answers for the interviewer to mark all the respondent’s answers (multiple options). The interviewers were instructed to ask the respondent “anything else?” repeatedly when the respondent stopped stating their responses, to explore the information in depth. There were open-ended questions and, for close-ended questions there were spaces for the interviewers to write all the responses that did not match the pre-defined options.

**Implementation of COVID-19 preventive measures at household level**—this included source of water to understand the presence and functioning of a handwashing facility, the capacity to keep a distance of 1.5 meters apart within the household premises, number of face masks in the household; and compliance with quarantine for members or visitors who had contact with suspected cases. The fieldworkers were instructed to observe/verify the existence and functioning of a hand washing facility in the household (presence of water, soap and/or ashes)—in line with international guidelines for collecting data on handwashing facilities [12]. Ashes were recommended by the government for handwashing when soap was not available [14].

**Symptoms of respiratory illness at household and community levels**–the household heads were asked whether they had had any symptom of respiratory illness since 05^th^ March 2020, when COVID-19 was first declared in South Africa; whether they or any member of the household had respiratory symptoms on the day of interview; and whether they knew about anyone with such symptoms in their neighbourhoods. The date when COVID-19 was first declared in South Africa was used as starting point because there were reports, in the media, of unprecedented inflow of Mozambicans returning from South Africa as the South African government had announced that it would close the borders due to COVID-19; and this inflow was seen as a potential source for importing Coronavirus into Mozambique [6].

**Perceived hardships due to COVID-19**—the respondents were asked “in this problem of Coronavirus, what worries you the most?” The interviewers would listen to the respondent for mentions of any of the pre-defined options and pick all that were mentioned. The pre-defined options included: “Hunger”; “Travel restrictions”; “I stopped working”; “I am not working well”; “Afraid of being infected myself”; “Afraid that some family member may be infected”; “Coronavirus does not have cure”; “I am afraid to lose my job”; “I have lost my job”; “Interruptions of classes”; and “others, specify”. Further, the survey asked whether there had been any social event that was postponed in the household due to Coronavirus.

### 2.5. Concepts and methods of data analysis

The key COVID-19 preventive measures were defined according to the *Manual de Prevenção da COVID-19* (Manual for the Prevention of COVID-19) published by the Ministry of Health of Mozambique in April 2020 [14], and other standard guidelines [15]. Thus, the wearing of face masks was defined as wearing a medical or non-medical mask by a person aged 6 years or more while indoor or outdoor settings where physical distancing of 1.5 meter minimum could not be maintained [16]. Social distancing was defined as keeping a minimum distance of 1.5 meter between people. Quarantine was defined as keeping someone who had a close contact with someone who had COVID-19 or was arriving from a place of risk of COVID-19, away from others for 14 days, including in their own home [17]. Confinement or staying at home was defined as making all the efforts to stay at home and avoid travelling, leaving home only in case of strong motives [14]. Frequent hand washing was defined as washing hands with water and soap or disinfecting with alcohol every time that a person has contact with objects or other items that have or may have been touched by somebody else [14].

The analysis presents descriptive statistics on perceptions and levels of compliance with COVID-19 preventive measures at individual and at household levels, comparing the two districts and comparing by other characteristics such as, occupation, age, level of education, and religion. T-test was performed to assess the statistical significance of the differences on proportions observed between Manhiça and Quelimane. The open-ended responses and the texts written to specify the “other” category were analysed using content analysis (resorting to Stata *regexm* function, which performs a match of a word or expression in a text, to arrange them in groups according to the main content of each text), to see if they could fit in any of the pre-defined responses or new categories needed to be created. Vague responses that could not permit assessing the accuracy of respondent’s knowledge and could not fit in any of the pre-defined categories were classified as “incomprehensive or vague answer”. Missing data and the “do not know” were treated and reported as such, but excluded when performing the T-test. The analyses were done using Stata 14.2 [18].

## 3. Results

### 3.1. Socio-demographic profile of participants and their households

The study interviewed 770 individuals, of which 62.3% were heads of households, 18.6%, their spouses, 11.0% their sons or daughters, and 8.1% others. Table 1 shows that the majority (65.7%) of the respondents were females, but there were more females in Manhiça (76.0%) than in Quelimane (55.4%). By education, 17.5% of participants were illiterate, while 43.4% had only primary education, and 39.1% had secondary or higher education. In relation to occupation, the majority (68.4%) were unemployed and were engaged on subsistence family activities such as farming, fishing, and production of fire wood/charcoal, followed by public sector officers and students (12.5%), and vendors in formal and informal sectors (9.5%). With regards to access to drinking water, 48.2% of households had their sources of water within the household premises, either piped water or wells, but Manhiça had higher percentage of households using piped water (59.6%) than Quelimane (36.8%).

**Table 1:** Socio-demographic characteristics of participants by study area.

Variable	Manhiça		Quelimane		Total	
	n	%	n	%	N	%
**Total of participants**	384	100	386	100	770	100
**Relation to household head**						
Head or spouse of head	307	79.9	316	81.9	623	80.9
Son or daughter	35	9.1	50	12.9	85	11.0
Other	42	10.9	20	5.2	62	8.1
**Sex of participant**						
Male	92	24.0	172	44.6	264	34.3
Female	292	76.5	214	55.4	506	65.7
**Age group in years**						
Adolescent (<19)	15	3.9	22	5.7	37	4.8
Adult (20–64)	317	82.6	345	89.4	662	86.0
Elder (65+)	52	13.5	19	4.9	71	9.2
**Level of education**						
None	93	24.2	42	10.9	135	17.5
Primary	196	51.0	138	35.8	334	43.4
Secondary or higher	95	24.7	206	53.4	301	39.1
**Religion**						
Protestant/Evangelic	203	52.9	43	11.1	246	32.0
Catholic	31	8.1	218	56.5	249	32.6
Zion/other/none	144	37.5	30	7.8	174	22.6
Islamic/Hindu	6	1.6	95	24.6	101	13.6
**Main occupation**						
Peasants/fisher/charcoal / do not work	285	74.5	242	62.7	527	68.4
Teachers/public officers / student	24	6.3	72	18.7	96	12.5
Vendors formal/informal	25	6.5	48	12.4	73	9.5
Carpenters/welding/builders	50	13.0	24	6.2	74	9.6
**Is your main source of water at home/yard**						
Yes	229	59.6	142	36.8	371	48.2
No	136	35.4	238	61.7	374	48.6
Missing	19	5.0	6	1.6	25	3.3
**Main source of water, independent of location**						
Piped water	263	68.4	202	52.6	465	60.4
Protected well	19	5.0	48	12.5	67	8.7
Unprotected well	30	7.9	84	21.8	114	14.9
Protected hole with pump	56	14.6	3	0.8	59	7.7
River, lake, rain, or other	9	2.3	37	9.7	46	6.0
Missing	7	1.8	11	2.8	18	2.3

### 3.2. Awareness about COVID-19 disease and its transmission dynamics

Table 2 presents data on the awareness of COVID-19 disease by key socio-demographic characteristics of participants. Nearly all (98.7%) study participants had heard of Coronavirus, but this percentage dropped to 89.2% when asked about COVID-19. The name COVID-19 was less known in Quelimane (86.0%) than in Manhiça (92.5%) (p = 0.004). Responses by religion did not differ between the two districts (p = 0.659), but they did by occupation–public sector officers and students appeared to be better informed than vendors and peasants/fisherman unemployed/retired (p = 0.005). In both Manhiça and Quelimane, the awareness about Coronavirus disease decreased with age and increased with level of education.

**Table 2:** Awareness about COVID-19 disease by socio-demographic characteristics of participants in Manhiça and Quelimane.

Variable	Manhiça		Quelimane		Total		Chi2 P-value*
	n	%	n	%	N	%	
**Total of participants**	384	100	386	100	770	100	
PANEL “A”							
**Have you ever heard of Coronavirus**							
Yes	379	98.7	381	98.7	760	98.7	0.712
No	3	0.8	4	1.0	7	0.9	
Missing	2	0.5	1	0.3	3	0.4	
**Have you ever heard of COVID-19**							
Yes	355	92.5	332	86.0	687	89.2	0.004
No	28	7.3	53	13.7	81	10.5	
Missing	1	0.3	1	0.3	2	0.3	
PANEL “B”							
**Awareness of Coronavirus by selected characteristics: % of those who said YES** **							
Total of respondents that said they had heard of Coronavirus**	**379**	**100**	**381**	**100**	**760**	**100**	
**Age group in years**							
Adolescent (<20)	15	100.0	22	100.0	37	100.0	0.906
Adult (20–64)	313	98.7	340	98.6	653	98.6	
Elder (65+)	51	98.1	19	100.0	70	98.6	
**Level of education**							
None	91	97.9	41	97.6	132	97.8	0.106
Primary	193	98.5	135	97.8	328	98.2	
Secondary and more	95	100.0	205	99.5	300	99.6	
**Religion**							
Catholic	31	100.0	215	98.6	246	98.8	0.659
Protestant/Evangelic	201	99.0	43	100.0	244	99.2	
Zion/none/others/none	141	97.9	29	96.7	170	97.7	
Islamic/Hindu	6	100.0	94	99.0	100	99.0	
**Main occupation**							
Peasants/fisher/unemployed	282	99.0	241	99.6	523	99.2	0.005
Vendors formal/informal	24	96.0	45	93.8	69	94.5	
Carpenters/welding/builders	49	98.0	23	95.8	72	97.3	
Public sector officers/ students	24	100.0	72	100.0	96	100.0	

Notes:

*—The P-value in Panel “A” and “B” refers to Pearson X^2^ test to see how significant the differences in responses between Manhiça and Quelimane are. Missing data and “do not know” were excluded in the calculation of X^2^, but we present them in the table to show their magnitude.

**—The focus on those who said YES to Coronavirus is because only 3 people said YES to both Coronavirus and COVID-19, so there is no need to repeat the tabulations for only 3 respondents.

Table 3 presents the different definitions of COVID-19 disease given by respondents, comparing Manhiça and Quelimane. More than a third (33.8%) of the respondents said they were unable to define this disease, 18.6% responded that it was a disease caused by a virus, 16.0%, it was a disease that causes cough, 14.8%, it was a respiratory disease; 9.0%, a dangerous, deadly and incurable disease; and 4.6%, a disease that comes from China.

**Table 3:** Participants’ definitions of COVID-19 disease in Manhiça and Quelimane.

Variable	Manhiça		Quelimane		Total		Chi^2^ P-value*
	n	%	n	%	N	%	
**What is Coronavirus or COVID-19?** **							
**Total of participants**	384	100	386	100	770	100	
Do not know	72	18.8	188	48.7	260	33.8	
Disease caused by a virus	30	7.8	113	29.3	143	18.6	0.000
Disease that causes cough	123	32.0	0	0.0	123	16.0	
Respiratory disease	97	25.3	17	4.4	114	14.8	
Dangerous/incurable/that kill	17	4.4	52	13.5	69	9.0	
Disease from China	26	6.8	9	2.3	35	4.5	
Missing	19	4.9	7	1.8	26	3.4	

Notes:

*—The P-value refers to Pearson X^2^ test to see how significant the differences in responses between Manhiça and Quelimane are. Missing data and “do not know” were excluded in the calculation of X^2^, but we present them in the table to show their magnitude.

**—Irrespective of having said YES or NO to the previous question that asked “have you ever heard of Coronavirus or COVID-19?” Originally, this question had been framed only for those who would say that they had heard of this disease, but the pilot phase showed that some of those who responded NO, would still say something in the definition. Thus, the interviewers were instructed to ask to all the participants.

Table 4 presents the responses on the knowledge of symptoms, means of transmission and prevention of Coronavirus, and it shows that 79.6% of respondents mentioned at least one symptom of Coronavirus, 77.4% mentioned at least one mean of transmission, and 84.8% mentioned at least one measure for preventing the disease. Manhiça had higher percentage of respondents who mentioned at least one symptom (84.4%) than Quelimane (74.9%), and of who mentioned at least one mean of transmission (78.9%) than Quelimane (75.9%). The number of symptoms mentioned ranged from 1 to 9; the number of means of transmission ranged from 1 to 5, and the number of prevention measures, from 1 to 8. About half of the respondents (49.3%) that mentioned at least one symptoms, indicated two to three symptoms, and 37.2% indicated four to five symptoms. The equivalent percentages for means of transmission were 60.9% and 21.5%, respectively. The comparable percentages for measures of prevention were 69.5% and 24.0%, respectively. However, some respondents were only able to mention one symptom (6.7%), one means of transmission (17.6%), and one measure of prevention (3.5%). The differences in these responses between Manhiça and Quelimane were generally statistically significant.

**Table 4:** Knowledge of symptoms, means of transmission and prevention of Coronavirus.

Variable	Manhiça		Quelimane		Total		Chi2 P-value*
	n	%	n	%	N	%	
**Total of participants**	**384**	**100%**	**386**	**100%**	**770**	**100%**	
**Participants that mentioned at least one symptom**							
Mentioned at least 1	324	84.4	289	74.9	613	79.6	0.206
Did not say anything	48	12.5	56	14.5	104	13.5	
Said did not know	12	3.1	41	10.6	53	6.9	
**Number of symptoms each respondent mentioned, if mentioned any**							
**Total**	**324**	**100.0**	**289**	**100.0**	**613**	**100.0**	
Two to three	166	51.2	136	47.1	302	49.3	0.000
Four to five	101	31.2	127	43.9	228	37.2	
Six and more	20	6.2	22	7.6	42	6.9	
One	37	11.4	4	1.4	41	6.7	
**Participants that mentioned at least one mean of transmission**							
Mentioned at least 1	303	78.9	293	75.9	596	77.4	0.254
Did not say anything	81	21.1	91	23.6	172	22.3	
Said did not know	0	0.0	2	0.5	2	0.3	
**Number of means of transmission each respondent mentioned, if any**							
**Total**	**303**	**100**	**293**	**100**	**596**	**100.0**	
Two to three	166	54.8	197	67.2	363	60.9	0.013
Four to five	76	25.1	52	17.7	128	21.5	
One	61	20.1	44	15.0	105	17.6	
**Participants that mentioned at least one mean of prevention**							
Mentioned at least 1	316	82.3	337	87.3	653	84.8	0.053
Did not say anything	68	17.7	49	12.7	117	15.2	
**Number of means of prevention each respondent mentioned, if any**							
**Total**	**316**	**100**	**337**	**100**	**653**	**100.0**	
Two to three	195	61.7	259	76.9	454	69.5	0.000
Four to five	89	28.2	68	20.2	157	24.0	
One	14	4.4	9	2.7	23	3.5	
Six and more	18	5.7	1	0.3	19	2.9	

Note:

*—The P-value refers to Pearson X^2^ test to see how significant the differences of responses between Manhiça and Quelimane are. Missing data and “do not know” were excluded in the calculation of X^2^, but we present them in the table to show their magnitude.

Table 5 presents details of which were the symptoms and means of transmission and prevention that were mentioned, and how many respondents mentioned which symptoms. The values by district tells how many respondents mentioned a specific symptom, out of the total of the district (based on multiple options-questions), e.g. 47.9% of the 384 participants in Manhiça and 52.1% out of the 386 in Quelimane mentioned dry cough, which means 50.0% out of the 770 study participants—the first column of total percentages. The second total column refers to times that a symptom was mentioned out of the total mentions in the two districts–it helps to see which symptoms were most mentioned out of the total mentions in the study sample. This table shows that dry cough (17.8%), fever (15.7%), flu-like symptoms (14.2%), breathing difficulties (13.6%), and pain in the throat (8.8%) were the symptoms most commonly mentioned by the respondents. The mechanisms of transmission most mentioned included touching an infected person or object (30.9%), inhaling the air from the mouth of an infected person (21.3%), contact with saliva droplets from an infected person (20.4%), and touching the mouth, nose or eyes (13.3%). The means of prevention most mentioned were washing hands with soap or alcohol (30.9%), using face mask always (28.6%), social distancing (16.6%), and avoiding crowded places (10.2%).

**Table 5:** Knowledge of symptoms, means of transmission and prevention of Coronavirus.

Variable	Manhiça		Quelim		Total			Chi2 P-value***
	n	*%* *	n	*%* *	N	*%* *	*%* **	
**What are the symptoms of Coronavirus? (multiple options)**								
Dry cough	184	47.9	201	52.1	385	50.0	17.8	0.249
Fever	149	38.8	191	49.5	340	44.2	15.7	0.003
Flu-like symptoms	183	47.7	124	32.1	307	39.9	14.2	0.000
Breathing difficulties	142	37.0	153	39.6	295	38.3	13.6	0.448
Head ache	124	32.3	159	41.2	283	36.8	13.1	0.010
Pain in the throat	113	29.4	76	19.7	189	24.5	8.7	0.002
Cough with sputum	58	15.1	68	17.6	126	16.4	5.8	0.346
Muscular pain	61	15.9	60	15.7	121	15.7	5.6	0.896
Don’t know	12	3.1	43	11.1	55	7.1	2.5	-
Vomiting	20	5.2	30	7.8	50	6.5	2.3	0.149
Other	7	1.8	7	1.8	14	1.8	0.6	0.992
Total multiple responses	1,053	-	1,112	-	2,165		100	-
**How is Coronavirus transmitted? (multiple options)**								
Touch infected person/object	240	62.5	248	64.3	488	63.4	30.9	0.615
Inhale air mouth of infected	186	48.4	150	38.9	336	43.6	21.3	0.007
Droplets of saliva of infected	169	44.0	153	39.6	322	41.8	20.4	0.219
Touch mouth or nose, eyes	106	27.6	104	26.9	210	27.3	13.3	0.837
Kiss infected person	100	26.0	61	15.8	161	20.9	10.2	0.000
Other transmission means	16	4.2	44	11.4	60	7.8	3.8	0.000
Do not know	0	0.0	2	0.5	2	0.3	0.1	-
Total of multiple responses	817	-	762	-	1,500		100.	-
**How can we prevent Coronavirus? (multiple options)**								
Wash hand/soap or alcohol	298	77.6	318	82.4	616	80.0	30.9	0.097
Use face mask always	275	71.6	296	76.7	571	74.2	28.6	0.108
Social distancing	155	40.4	177	45.9	332	43.1	16.6	0.124
Avoid crowded places	124	32.4	80	20.7	204	26.5	10.2	0.000
Avoid touch mouth/noise/eye	57	14.8	53	13.7	110	14.3	5.5	0.659
Other means	21	5.5	42	10.9	63	8.2	3.2	0.006
Quarantine	31	8.1	23	6.0	54	7.0	2.7	0.251
Avoid travelling	42	10.9	3	0.8	45	5.8	2.3	0.000
Total multiple responses	1,003	-	992	-	1,995		*100*.	-

Notes:

*—Percentage of respondents that mentioned a specific symptom–(how many respondents mentioned a specific symptom, out the total of the district, and out of the total study sample).

**—Percentage of times that a symptom was mentioned–tells which symptom or means of transmission were most mentioned out of the total mentions in the study sample.

***—The P-value refers to Pearson X^2^ test to see how significant the differences in responses between Manhiça and Quelimane are. Missing data and “do not know” were excluded in the calculation of X^2^, but we present them in the table to show their magnitude.

### 3.3. Knowledge and perceptions of COVID-19 preventive measures

Table 6 presents the results on how people understood the key COVID-19 preventive measures assessed in this study. The majority of respondents knew that social distancing refers to keeping some distance between each other (62.0%) [62.0% results from the addition of “keeping 1.5–2 meters from others” (43.9%) and “to be away/be distant from others” (18.1%)], but 19.9% provided incomprehensive or vague answers, and 17.5% said that they did not know. In relation to hand washing in the context of COVID-19, 32.9% of respondents gave vague responses, 28.6% said that they washed their hands when leaving or arriving at home; 26.0%, after touching something or someone; and 7.7%, after using the toilet or when the hands were dirty or when they were about to eat something. Quarantine was defined as staying at home or in the same place by 36.6% of respondents; 26.5% defined it comprehensively as isolating someone who was sick or suspected or who had contact with positive or suspected cases (some respondents in this group indicated the period of 14 days); but 29.2% said that they did not know what it was. In relation to avoiding crowded places, two thirds of participants (60.0%) mentioned that it meant avoiding places with many people (60.0% results from the addition of 25.1%, 18.1%, 10.4% and 6.5%), but 27.0% of respondents gave inconclusive or astray answers, and 9.5% said that they did not know what it meant. With regards to avoiding travelling, 64.8% of respondents could not give a comprehensive response, 16.4% said that they did not know what it meant, 10.8% said it meant to stay at home, and 5.7% said it meant to travel only for extreme cases.

**Table 6:** Participants’ understanding of anti-COVID-19 measures.

Variable	Manhiça		Quelimane		Total		Chi2 P-value*
	n	%	n	%	N	%	
**Total of participants**	384	100	386	100	770	100	
**What social distancing means?**							
To keep 1,5-2m from others	170	44.3	168	43.5	338	43.9	*0*.*000*
To be away/distant from other	37	9.6	102	26.4	139	18.1	
Inconclusive/astray answer	93	24.2	60	15.5	153	19.9	
Don’t know	79	20.6	56	14.5	135	17.5	
Missing	5	1.3	0	0.0	5	0.6	
**In which situations do you wash your hands, in this context of COVID-19**							
Inconclusive/astray answer	173	45.1	80	20.7	253	32.9	*0*.*000*
When leaving/returning home	47	12.2	173	44.8	220	28.6	
After touching something/one	116	30.2	84	21.8	200	26.0	
After toilet/before meals/dirty	14	3.6	45	11.7	59	7.7	
Don’t know	24	6.3	3	0.8	27	3.5	
Missing	10	2.6	1	0.3	11	1.4	
**What it means to avoid crowded places?**							
Inconclusive/astray answer	113	29.4	95	24.6	208	27.0	*0*.*000*
Places more than 5–10 people	90	23.4	103	26.7	193	25.1	
Places with many people	30	7.8	109	28.2	139	18.1	
Places with 20 +people	57	14.8	23	6.0	80	10.4	
Don’t know	31	8.1	42	10.9	73	9.5	
Places with 50+ people	43	11.2	7	1.8	50	6.5	
Missing	20	5.2	7	1.8	27	3.5	
**What it means to avoid travelling?**							
Inconclusive/astray answer	208	54.2	291	75.4	499	64.8	*0*.*000*
Don’t know	67	17.4	59	15.3	126	16.4	
To stay at home	58	15.1	25	6.5	83	10.8	
To travel only for extreme case	42	10.9	2	0.5	44	5.7	
Missing	9	2.3	9	2.3	18	2.3	
**What it means to quarantine?**							
To stay at home or same place	121	31.5	161	41.7	282	36.6	*0*.*000*
Don’t know	112	29.2	113	29.3	225	29.2	
To isolate sick/suspect person	127	33.1	77	19.9	204	26.5	
Inconclusive/astray answer	14	3.6	25	6.5	39	5.1	
Missing	4	1.0	10	2.6	14	1.8	
**What it means to avoid touching the mouth, or eyes, or nose**							
Inconclusive/astray answer	112	29.2	210	54.4	322	41.8	*0*.*000*
Wash hands before touching them	147	38.3	60	15.5	207	26.9	
Don’t know	72	18.8	83	21.5	155	20.1	
Missing	53	13.8	33	8.5	86	11.2	

Note:

*—The P-value refers to Pearson X^2^ test to see how significant the differences of responses between Manhiça and Quelimane are. Missing data and “do not know” were excluded in the calculation of X^2^, but we present them in the table to show their magnitude.

### 3.4. Implementation of anti-COVID-19 measures at individual level

Table 7 presents the results of participants’ self-assessment of their level of compliance with anti-COVID-19 measures. It shows that wearing face masks in public places was the measure that more people (48.8%) said that they complied with it in all (100%) of the occasions, followed by avoiding unnecessary travels (40.0%), avoiding crowed places (34.0%), social distancing outside the household (34.4%), hand washing (21.9%), and staying at home (20.0%). On the other hand, the most difficult anti-COVID-19 measure was avoiding touching the mouth or nose or eyes, with 28.7% of respondents saying that they did not comply with it at all, and 48.7% complied only in 50% or less of the occasions they thought it was necessary—leading to a total of 77.4% respondents who said that they complied in 50% to 0% of the occasions. This was followed by social distancing within the household (73.1%), staying at home (66.2%) and frequent hand washing (56.0%). By districts, Manhiça had more respondents indicating higher degree of compliance with these measures than Quelimane (p = 0.000 for all the measures).

**Table 7:** Self-assessment of compliance with anti-COVID-19 measures at individual level.

Variable	Manhiça		Quelimane		Total		Chi2 P-value*
	n	%	n	%	N	%	
**Total of participants**	384	100	386	100	770	100	
**Degree of compliance with staying at home**							
0%	153	39.8	123	31.9	276	35.8	0.000
100%	98	25.5	56	14.5	154	20.0	
25%	57	14.8	63	16.3	120	15.6	
50%	44	11.5	70	18.1	114	14.8	
75%	23	6.0	74	19.2	97	12.6	
**Degree of compliance with hand-washing always**							
50%	132	34.4	94	24.4	226	29.4	0.000
25%	53	13.8	119	30.8	172	22.3	
100%	107	27.9	62	16.1	169	21.9	
75%	87	22.7	80	20.7	167	21.7	
0%	2	0.5	31	8.0	33	4.3	
**Degree of compliance with face mask wearing in public places**							
100%	192	50.0	184	47.7	376	48.8	0.000
50%	85	22.1	87	22.5	172	22.3	
75%	70	18.2	71	18.4	141	18.3	
25%	32	8.3	39	10.1	71	9.2	
0%	3	0.8	5	1.3	8	1.0	
**Degree of compliance with avoiding touching the month, nose, and eyes**							
0%	18	4.7	203	52.6	221	28.7	0.000
25%	131	34.1	62	16.1	193	25.1	
50%	129	33.6	53	13.7	182	23.6	
75%	52	13.5	60	15.5	112	14.5	
100%	52	13.5	7	1.8	59	7.7	
**Degree of compliance with social distancing within the household**							
0%	18	4.7	192	49.7	210	27.3	0.000
25%	131	34.1	51	13.2	182	23.6	
50%	129	33.6	42	10.9	171	22.2	
75%	52	13.5	50	13.0	102	13.2	
100%	52	13.5	7	1.8	59	7.7	
**Degree of compliance with social distancing outside the household**							
75%	111	28.9	154	39.9	265	34.4	0.000
100%	138	35.9	85	22.0	223	29.0	
50%	94	24.5	93	24.1	187	24.3	
25%	31	8.1	37	9.6	68	8.8	
0%	8	2.1	17	4.4	25	3.2	
**Degree of compliance with avoiding crowed places**							
100%	157	40.9	105	27.2	262	34.0	0.000
75%	96	25.0	128	33.2	224	29.1	
50%	94	24.5	99	25.6	193	25.1	
25%	30	7.8	33	8.5	63	8.2	
0%	5	1.3	21	5.4	26	3.4	
**Degree of compliance with avoiding unnecessary travels**							
100%	208	54.2	100	25.9	308	40.0	0.000
50%	68	17.7	99	25.6	167	21.7	
75%	79	20.6	62	16.1	141	18.3	
25%	19	4.9	95	24.6	114	14.8	
0%	8	2.1	30	7.8	38	4.9	

Note:

*—The P-value refers to Pearson X^2^ test to see how significant the differences in responses between Manhiça and Quelimane are.

### 3.5. Implementation of anti-COVID-19 measures at household level

Table 8 presents the degree of compliance with anti-COVID-19 measures at household level, assessed by combining data from questions and observations, as described in the section on methods. It shows that sixty-nine per cent (69.4%) of households did not have hand washing facilities or disinfectants (75.6% in Quelimane and 63.0% in Manhiça); and of those that did have devices, 58.5% had no water in the hand washing facility, 62.0% had no soap, and 74.4% had no ash in these devices. In addition to hand washing facilities, it was asked whether there was any other disinfectant or not– 22.6% had at least one other disinfectant such as alcohol or alcohol gel. The other indicator was the existence and the number of masks in the household—almost all households (98.1%) had masks, but the ratio of masks per household member was very low (median of 1 mask per member aged 6+ years).

**Table 8:** Implementation of anti-COVID-19 measures at household level, Manhiça and Quelimane.

Variable	Manhiça		Quelimane		Total		Chi2 P-value*
	n	%	n	%	N	%	
**Total of households**	384	*49*.*9*	386	*50*.*1*	770	*100*	
**Is there a hand washing facility in the household?**							
Yes	141	36.7	93	24.1	234	30.4	
No	242	63.0	292	75.6	534	69.4	0.001
Missing	1	0.3	1	0.3	2	0.3	
Total households with washing	**141**	***100*.*0***	**93**	***100*.*0***	**234**	***100*.*0***	
**Is there water in the hand washing facility?**							
Yes	44	31.2	52	55.9	96	41.0	
No	96	68.1	41	44.1	137	58.5	0.001
Missing	1	0.7	0	*0*.*0*	1	0.4	
**Is there soap in the hand washing facility?**							
Yes	44	31.2	44	47.3	88	37.6	
No	97	68.8	48	51.6	145	62.0	0.018
Missing	0	0.0	1	1.1	1	0.4	
**Is there ash in the hand washing facility?**							
Yes	19	13.5	34	36.6	53	22.6	
No	116	82.3	58	62.4	174	74.4	0.000
Missing	6	4.3	1	1.1	7	3.0	
**Is there any other disinfectant in the hand washing facility?**							
Yes	19	13.5	34	36.6	53	22.6	
No	93	66.0	58	62.4	151	64.5	0.000
Missing	29	20.6	1	1.1	30	12.8	
**Face masks in the household**							
Median of masks p/ household	4		5		5		
Household without face masks	5		10		15		
Ratio of face mask/person 6+y	1.1		0.9		0.9		

Note:

*—The P-value refers to Pearson X^2^ test to see how significant the differences in responses between Manhiça and Quelimane are. Missing data and “do not know” were excluded in the calculation of X^2^, but we present them in the table to show their magnitude.

### 3.6. Prevalence of symptoms of respiratory illness

The results on the prevalence of symptoms of respiratory illness show that 93 (12.1%) respondents have had symptoms sometime since COVID-19 was announced first in South Africa (05 March 2020), and 38.1% of them had two or more episodes of such symptoms. However, only 6 households (0.8%) had a person with symptoms at the date of interview, of which 4 had only one person and 2 had two people with symptoms. By district, Quelimane had significantly higher number of (past and current) cases with symptoms 80 (20.7% of respondents) than Manhiça 13 (3.4%), (p = 0.000).

### 3.7. Perceived hardships due to Coronavirus

Closure of educational institutions was the COVID-19 related leading hardship perceived by the respondents (with 18.3% of mentions), followed by hunger (17.5%), fear of being infected or having a family member infected (12.2%), and other worries (12.0%), (Table 9). The “other worries” included fear of death and of uncertainty of the future (32.1%), closure of churches (28.3%), and loss of opportunities for employment and other means of livelihood (18.3%). Most of these hardships were reported with significant differences by district.

**Table 9:** Participants’ worries caused by Coronavirus in Manhiça and Quelimane.

Variable	Manhiça		Quelimane		Total			Chi2 P-value***
	n	%*	n	%*	N	%*	%**	
**Total of participants**	384	100	386	100	770	100		
**In this context of Coronavirus, what worries you the most? (multiple options)**								
Closure of schools	200	52.1	134	34.7	334	43.4	18.3	0.000
Hunger	155	40.4	164	42.5	319	41.4	17.5	0.550
Me or family member be infected	34	8.9	189	49.0	223	29.0	12.2	0.000
Other worries	96	25.0	123	31.9	219	28.4	12.0	0.035
I am not working well	97	25.3	70	18.1	167	21.7	9.2	0.016
Travel restrictions	110	28.6	54	14.0	164	21.3	9.0	0.000
COVID-19 has no cure	96	25.0	45	11.7	141	18.3	7.7	0.000
Increasing numbers in Moz. & out	57	14.8	77	19.9	134	17.4	7.4	0.062
I lost job or stopped working	39	10.2	47	12.2	86	11.2	4.7	0.374
I am afraid of losing my job	31	8.1	4	1.0	35	4.5	1.9	0.000
Total of times mentioned	915	-	907	-	1,822		100.0	-
**Other worries in the context of Coronavirus (if option not in the list above)**								
Afraid death & uncertain future	14	3.6	54	14.8	71	9.2	32.4	0.000
Closure of churches	41	10.7	21	5.4	62	8.1	28.3	0.000
Loss of job & econ. opportunities	26	6.8	14	3.6	40	5.2	18.3	0.003
Others/Don’t know	12	3.1	13	4.1	28	3.6	12.8	
Increase of prices	1	0.3	12	3.4	14	1.8	6.4	0.004
Missing	2	0.5	2	0.5	4	0.5	1.8	
Total	96	-	123	-	219		100.0	-
**Was there any social events that was postponed in the household due to Coronavirus**								
Yes	76	19.8	119	30.8	195		25.3	0.001
No	300	78.1	267	68.2	567		73.6	
Missing	8	2.1	0	0.0	8		1.0	
**Social events that were postponed in the household due to Coronavirus (multiple options)**								
Other events	9	2.3	71	18.4	80	10.4	33.6	0.000
Wedding and similar ceremonies	16	4.2	40	10.4	56	7.3	23.5	
Religious/traditional ceremony	39	10.2	15	3.9	54	7.0	22.7	
Birth date party	14	3.6	18	4.7	32	4.2	13.4	
*Xitique* (family/friends’ gathering)	8	2.1	8	2.1	16	2.1	6.7	
Total	86		152		238		100.0	

Notes:

*—Percentage of respondents that mentioned a specific problem–tells how many respondents mentioned a specific problem out of the total of district, and out of the total study sample).

**—Percentage of times that a problem was mentioned–tells which problems were most mentioned out of the total mentions in the study sample.

***—The P-value refers to Pearson X^2^ test to see how significant the differences in responses between Manhiça and Quelimane are. Missing data and “do not know” were excluded in the calculation of X^2^, but we present them in the table to show their magnitude.

In relation to postponement of social events due to Coronavirus, the data show that 25.3% of respondents said that they had postponed at least one social event in their households– 31.8% of these households postponed more than one event. Wedding and similar ceremonies (23.5%), religious and traditional ceremonies (22.7%), birthday parties (13.4%), and gatherings for *xitique* (6.7%) were the leading postponed events. *Xitique* is a term in local language of Southern Mozambique (Changana), but also used equally in Quelimane, for a network or group of friends, family members, or colleagues for inter-support or other means of solidarity involving money saving which is rotatively paid to one member of the group. Often it involves social gathering in the household of the member that is receiving the group’s savings [19,20].

## 4. Discussion

This study aimed to assess community perceptions and levels of compliance with anti-COVID-19 measures recommended by the government of Mozambique in the districts of Manhiça and Quelimane. The findings revealed nearly universal awareness (98.7%) of the term Coronavirus disease, and a slightly lower awareness (89.2%) of the term COVID-19 specifically. Although a majority of participants were able to provide sufficient definitions of COVID-19 based on its symptoms and causes, a considerable portion (one in three participants) said they were unable to define this disease. In terms of comprehending the symptoms, means of transmission and prevention of Coronavirus, the communities demonstrated elevated levels of awareness. Nevertheless, differences emerged by districts, with Manhiça exhibiting stronger awareness compared to Quelimane.

During the conception and execution of this study, numerous challenges emerged, spanning conceptual, operational, and safety considerations. Notably, concerns loomed over the potential exposure of both researchers and participants to the highly contagious and life-threatening disease under investigation. In relation to bias, we believe that some participants may have provided socially desirable responses [21], during the interviews due to the fact that the interviewers came from a health research institution known to the communities both in Manhiça and in Quelimane. However, no adjustments for errors or for confounders were performed in this study; and no assessment of the magnitude or the direction of potential bias was performed. The study’s approach of using both multiple-choice and open-ended questions, coupled with interviewers being directed to prompt respondents with "anything else?" to capture additional information until exhaustively stated, can be viewed as a strength of this study. This strategy facilitated the exploration of extensive information and the capture of maximum response variability.

Although this study indicated higher proportions of participants exhibiting awareness of Coronavirus and fair level of comprehension regarding certain anti-COVID-19 measures, there were notably elevated percentages of responses characterized as vague or inconclusive when asked to define these key measures. These ranged from 5.1% in the context of defining "quarantine" to a more substantial 64.8% when defining the concept of "avoiding traveling." Incorporating the "do not know" responses into these ambiguous answers leads to the deduction that a substantial portion of respondents lacked sufficient comprehension of the meanings behind many of the anti-COVID-19 measures. During that period, numerous novel concepts emerged in relation to the pandemic, and the terms "Coronavirus," "Corona," and "COVID-19" seemed to be employed interchangeably to refer to the same thing. Likewise, even some apparently obvious terms like confinement, stay at home, immobility, and quarantine had not been well assimilated by the general public in Mozambique, coupled with the practical challenges around their implementation. In the later months of 2020, a new term emerged in public discourse, namely “the new normal”–intended to raise awareness and sensitize the population to adjust to this new pandemic context for longer periods of time. However, certain individuals misinterpreted it, mistakenly believing that it implied a return to pre-pandemic normal daily life. This miss-interpretation may have been influenced by the fact that the government was relaxing some of the stringent anti-COVID-19 measures. Studies conducted in Mozambique during the time of our survey, underscored the importance of addressing misinformation about Coronavirus, presenting a noteworthy concern for policymakers. Illustratively, a study conducted by PERC [22] in Maputo city, brought forth compelling findings. Within this study, a substantial 72% of respondents held the belief that hot climates could deter COVID-19, while 41% subscribed to the notion that COVID-19 was a product of government-engineered biological warfare. Additionally, 39% expressed a desire for more comprehensive information, particularly regarding COVID-19 protection, its origins, and potential remedies. Another study showed that some respondents in rural and urbans areas of Central Mozambique did not believe that COVID-19 existed, and others said that fighting for food was of high priority than following anti-COVID-19 measures [23].

Beyond misinformation, the challenge of comprehending COVID-19-related messages could stem from the rapidly evolving nature of these governmental directives. Addressing this weakness is imperative, not solely concerning the public’s perspective, but also regarding how these measures were defined by the government itself. For instance, the concept of maintaining distance initially circulated as "social distancing," later shifting to "physical distancing," with the recommended minimum gap initially ranging from 1 to 1.5 meters before being updated to 2 meters (although the latter was less frequently disseminated). This dynamic was mirrored in the classification of "crowded places," contingent on whether gatherings were indoors or outdoors, subsequently influencing the designated maximum capacity. Furthermore, this capacity diverged among contexts, including funeral gatherings, which saw consistent fluctuations in attendees allowances. The constantly changing and at times unclear measures likely exacerbated the confusion within the population.

Compliance with anti-COVID-19 measures at individual and at household levels was low, both in Manhiça and in Quelimane–below 50% in most of the indicators used in this study. Even the apparently easiest measure (mask wearing in public places), was only followed by half of the respondents, in spite of acknowledging that they should be doing it, and there was a lack of sufficient masks in the households. A similar result (55.5% of overall level of compliance) was reported in a study on compliance with COVID-19 preventive measures among food and drink establishments in Ethiopia, in 2020 [24]. Our study found that only 40.0% of the respondents said that they were avoiding unnecessary travels in 100% of the times, 34.0% were avoiding crowed places, and 29.1% were doing social distancing outside the household. Structural and personal factors may have contributed to lower compliance as most people live on daily-income activities and even those with a monthly income had to go for work using crowed public transports.

The prevalence of symptoms of respiratory illness in the households was very low, and most respondents did not know anyone with such symptoms in their communities. However, the meaning or usefulness of these findings must be taken cautiously because of the bias involved in self-reporting of symptoms, when compared to medically diagnosed disease, particularly because some studies indicated that 80% of infected people in Mozambique were asymptomatic [14]–a percentage similar to what was reported in India (75%) in 2020 [25].

With regards to hardships imposed to people and their households by the pandemic, this study found that the closure of educational institutions, fear of being infected, hunger, fear of death and of uncertainty of the future, and the closure of churches were the main hardships–and these responses seem expected, particularly for Manhiça and Quelimane where little or no social services exist for the most pre-COVID-9 vulnerable populations, whose suffering has increased due to Coronavirus. A study in Philippines on how COVID-19 impacted vulnerable communities, reported increased lack of income opportunities and insufficient food supply that existed before COVID-19 but had worsened due to the pandemic [26].

## 5. Conclusions

Our findings demonstrated that community members exhibited substantial awareness of Coronavirus, knowledge of the disease, including its transmission and preventive dynamics. However, the level of understanding of anti-COVID-19 measures was notably deficient, which suggests that the pandemic-related messages might not have been effectively conveyed in an informative manner that resonated within the general population. A deeper exploration of the factors influencing the effective implementation of these measures is required for informing decision-makers about ways to improve community knowledge and practices to prevent infectious diseases with epidemic potential.

## References

[1] Cucinotta D. and Vanelli M., “WHO Declares COVID-19 a Pandemic.,” *Acta Biomed*, vol. 19, no. 1, pp. 157–160, 2020, doi: 10.23750/abm.v91i1.9397 32191675 PMC7569573

[2] WHO, “The World Health Organization and Wikimedia Foundation expand access to trusted information about COVID-19 on Wikipedia,” 2020. https://www.who.int/news/item/22-10-2020-the-world-health-organization-and-wikimedia-foundation-expand-access-to-trusted-information-about-covid-19-on-wikipedia

[3] Bedford J. et al., “COVID-19: Towards controlling of a pandemic,” *Lancet*, vol. 395, no. 10229, pp. 1015–1018, Mar. 2020, doi: 10.1016/S0140-6736(20)30673-5 32197103 PMC7270596

[4] da Republica A., *Boletim da Republica*: *Decreto Presidencial n*.*o 11/2020*, *de 30 de Março que Decreta o Estado de Emergência*. Maputo, 2020, pp. 0–1.

[5] MISAU, “Plano Nacional de Preparação e Resposta a Pandemia do Covid-19,” Maputo, 2020. [Online]. https://www.misau.gov.mz/index.php/covid-19-em-mocambique-relatorios?download=1252:relatorio-do-1-ano-2020-2021-covid-19-em-mocambique

[6] Lusa, “Vendedores informais de Maputo têm de escolher entre a fome e a doença; 31 de Marco 2020,” Mar. 31, 2020. [Online]. https://www.rtp.pt/noticias/covid-19/vendedores-informais-de-maputo-tem-de-escolher-entre-a-fome-e-a-doenca_n1217054.

[7] Dahab M. et al., “COVID-19 control in low-income settings and displaced populations: What can realistically be done?,” *Confl*. *Health*, vol. 14, no. 1, 2020, doi: 10.1186/s13031-020-00296-8 32754225 PMC7393328

[8] Arnaldo C., “Fertility and its proximate determinants in Mozambique: an analysis of levels, trends, differentials and region variation,” Australian National University, Sidney, 2003.

[9] Munguambe K. et al., “Consent to minimally invasive tissue sampling procedures in children in Mozambique: A mixed-methods study,” *PLoS One*, vol. 16, no. 11, p. e0259621, Nov. 2021, doi: 10.1371/journal.pone.0259621 34748582 PMC8575303

[10] I. N. de E. INE, “QUADRO 1. POPULAÇÃO RECENSEADA POR ÁREA DE RESIDÊNCIA E CATEGORIA CENSITÁRIA, SEGUNDO SEXO E IDADE. PROVÍNCIA DA ZAMBEZIA, 2017,” 2022. http://www.ine.gov.mz/iv-rgph-2017/zambezia/quadro-1-populacao-recenseada-por-area-de-residencia-e-categoria-censitaria-segundo-sexo-e-idade-provincia-da-zambezia-2017.xlsx/view.

[11] Nhacolo A. et al., “Cohort profile update: Manhiça Health and Demographic Surveillance System (HDSS) of the Manhiça Health Research Centre (CISM),” *Int*. *J*. *Epidemiol*., pp. 1–8, Jan. 2021, doi: 10.1093/ije/dyaa218 33452521 PMC8128467

[12] WHO, UNICEF, and JMP, *Progress on Drinking Water*, *Sanitation and Hygiene: 2017*, vol. 33, no. 11. 2017. doi: 10.1016/0278-6915(95)00065-A 7590541

[13] Fleiss J. L., Levin B., and Paik M. C., *Statistical Methods for Rates and Proportions*. Hoboken, NJ, USA: John Wiley & Sons, Inc., 2003. doi: 10.1002/0471445428

[14] MISAU, *Manual de prevencao da COVID-19*. Maputo, 2020. [Online]. https://www.misau.gov.mz/index.php/manuais-e-material-educativo?download=322:manual-de-prevencao-a-covid-19.

[15] CDC, “Handwashing in Communities: Clean Hands Save Lives,” 2020. https://www.cdc.gov/handwashing/index.html.

[16] WHO, “Mask use in the context of COVID-19,” *World Heal*. *Organ*., no. December, pp. 1–10, 2020, [Online]. https://www.who.int/publications/i/item/advice-on-the-use-of-masks-in-the-community-during-home-care-and-in-healthcare-settings-in-the-context-of-the-novel-coronavirus-(2019-ncov)-outbreak.

[17] CDC, “COVID-19: Quarantine vs. Isolation,” p. 317422, 2020.

[18] StataCorp, “Stata 14.2 Statistics/Data Analysis.” StataCorp LP, Texas, 2015.

[19] C. C. Trindade, “Convívio e solidariedade: práticas de xitique em Moçambique,” 2011. [Online]. https://silo.tips/download/convivio-e-solidariedade-praticas-de-xitique-em-moambique.

[20] e Silva T. C., “A organização dos trabalhadores do sector informal dos mercados de Maputo e sua acção na promoção de melhores condições de vida e de trabalho O papel da Associação dos Operadores e Trabalhadores do Sector Informal—ASSOTSI,” Genebra, 2005. [Online]. Available: https://www.ilo.org/wcmsp5/groups/public/—europe/—ro-geneva/—ilo-lisbon/documents/publication/wcms_714572.pdf.

[21] Bogner K. and Landrock U., “Response Biases in Standardised Surveys,” Mannheim, Germany, 2016. doi: 10.15465/gesis-sg_en_016

[22] PERC, “Effective Implementation of Public Health and Social Measures in Mozambique: Situational Analysis,” 2020. [Online]. https://preventepidemics.org/wp-content/uploads/2020/05/Mozambique_perc-countrybrief_mobility.pdf.

[23] Chaimite L. P. E., “Perceptions of COVID-19 in Mozambique and the influence of ‘intermediaries,’” *Institute of Development Studies*, 2020. https://www.ids.ac.uk/opinions/perceptions-of-covid-19-in-mozambique-and-the-influence-of-intermediaries/.

[24] Kayrite Q. Q., Hailu A. A., Tola T. N., Adula T. D., and Lambyo S. H., “Compliance with COVID-19 Preventive and Control Measures among Food and Drink Establishments in Bench-Sheko and West-Omo Zones, Ethiopia, 2020,” *Int*. *J*. *Gen*. *Med*., vol. Volume 13, pp. 1147–1155, Nov. 2020, doi: 10.2147/IJGM.S280532 33235488 PMC7680164

[25] Dayanand D. et al., “Community seroprevalence and risk factors for SARS-CoV-2 infection in different subpopulations in Vellore, India, and their implications for future prevention,” *Int*. *J*. *Infect*. *Dis*., vol. 116, pp. 138–146, Mar. 2022, doi: 10.1016/j.ijid.2021.12.356 34971822 PMC8712712

[26] Fallesen D., “How COVID-19 impacted vulnerable communities in the Philippines,” 2021. https://blogs.worldbank.org/eastasiapacific/how-covid-19-impacted-vulnerable-communities-philippines.

